# Computer aided analysis of disease linked protein networks

**DOI:** 10.6026/97320630015513

**Published:** 2019-07-31

**Authors:** Soudabeh Sabetian, Mohd Shahir Shamsir

**Affiliations:** 1 Department of Biological and Health Sciences, Faculty of Bioscience and Medical Engineering, Universiti Teknologi Malaysia, 81310 Johor, Malaysia; 2 Infertility Research Center, Shiraz University, Shiraz 71454, Iran, Shiraz University of Medical Sciences, Shiraz, Iran

**Keywords:** Protein interaction network, disease network, computational method

## Abstract

Proteins can interact in various ways, ranging from direct physical relationships to indirect interactions in a formation of protein-protein interaction network. Diagnosis of the protein connections is critical to identify various cellular pathways. Today constructing and analyzing the protein interaction network is being developed as a powerful approach to create network pharmacology toward detecting unknown genes and proteins associated with diseases. Discovery drug targets regarding therapeutic decisions are exciting outcomes of studying disease networks. Protein connections may be identified by experimental and recent new computational approaches. Due to difficulties in analyzing in-vivo proteins interactions, many researchers have encouraged improving computational methods to design protein interaction network. In this review, the experimental and computational approaches and also advantages and disadvantages of these methods regarding the identification of new interactions in a molecular mechanism have been reviewed. Systematic analysis of complex biological systems including network pharmacology and disease network has also been discussed in this review.

## Background

**Figure 1:**
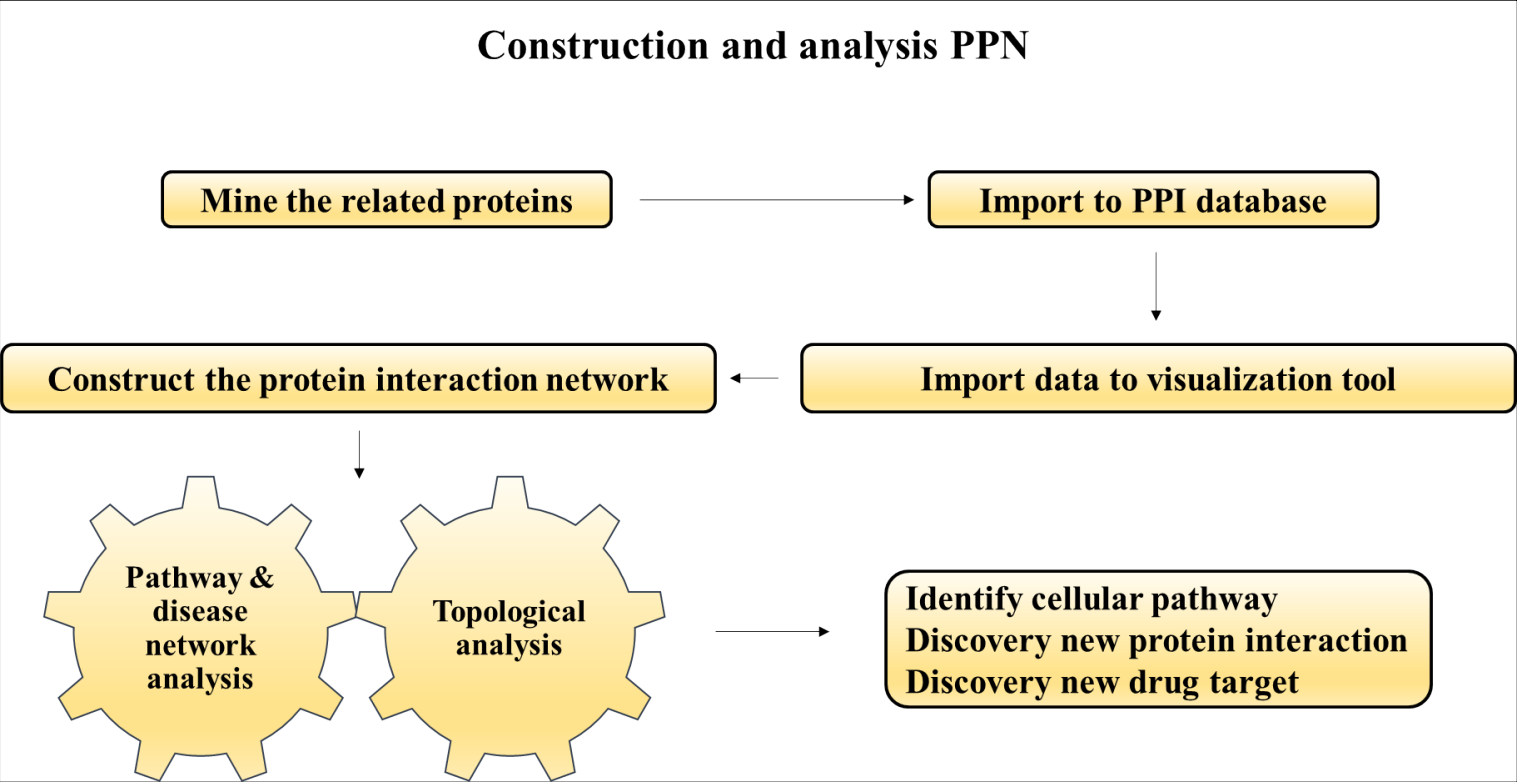
Graphical abstract for the study

The links between proteins in a protein set can make a proteinprotein interaction network (PIN) and all proteins that are involved in PINs are spatially or temporally engaged to interact with other proteins within the process as well as functioning as indirect interacting members of the same pathway [1].Currently, the discovery of protein connections has been assisted by developments in both biochemical and computational methods, which have produced precious awareness into the fundamental building of protein interactions in cellular networks [2].Until now, the original experimental methods, for instance, mass spectrometry and proteomics approaches, have been used to identify proteinprotein interaction [3]. However, due to the complexity of the problems of analyzing in-vivo PPIs such as protein denaturing and possible disruption in the formation of protein complexes, many efforts have failed to comprehensively elucidate the molecular interactions that facilitate molecular mechanism [4]. Existing computational approaches, in addition to experimental methods, can assist our understanding of PPIs at various levels. The computational approaches may be utilised for comprehensive examination or perform a wide scale analysis across large datasets (Figure 1).This approach signifies the multifaceted association of proteins with PPI links in a protein interaction network and would help to comprehend how signalling pathways linked with a disease are connected [5]. By using computational methods, it is possible to identify comprehensive information about complex diseases such as the putative disease target genes, putative drug targets, new prognostic biomarkers and understanding the mechanism of complex disease by network analysis [6]. Thus, computational approach denotes a novel technique for investigating the complex impacts of candidate genes that are connected to complex diseases, and is also worthwhile in recognizing important drug targets and genes in a disease and in complex biological systems [7].

## Experimental methodologies to detect PPI

Proteins commonly do not perform alone, but conduct their job in assisting other proteins and all cellular processes that depend on protein-protein interactions (PPIs) [8]. Therefore, identifying and characterizing PPIs is essential for understanding life at a molecular level. In order to understand the cellular and protein function at molecular level, knowledge of PPIs is critical. Many experimental methods exist to detect protein-protein interactions [9]. PPIs were recognized by utilizing hypothesis driven and the top-down methods of biophysics, biochemistry and genetics. New bottom-up interaction discovery methods with the progresses in proteomics technology, for instance 2D gel electrophoresis associated with the mass spectrometry and the yeast two-hybrid system, have been established to discover protein interactions at large scale. In numerous large-scale protein- protein interaction datasets, experimentally tested interactions have been assembled [10]. The genetically engineered strains of yeast (Saccharomyces cerevisiae) are employed by the Yeast Two-Hybrid (Y2H) system in order to classify protein-protein interaction. In order to discover interactions through the whole proteome of an organism, the Y2H is dominant method that can be applied in a high-throughput mode. It has been employed to identify proteome varied interactions in model organisms, for instance S. cerevisiae, H. pylori, D. melanogaster and C. elegans [11].

The main disadvantages of this Y2H technique are: it allows to analyze, two proteins at the time, the many proteins that are not in their native state as it occurs in the nucleus and the interactions do not take into account the physiological setting [12]. Mass spectrometry utilizes specific proteins what are tagged as "hooks" to refine biochemically whole protein complexes, then the purified proteins will be separated and their components identified by mass spectrometry [13].The benefits of utilizing this detection technique are that numerous members of a complex can be tagged, providing an internal stability check and it identifies protein complexes in their physiological condition. In contrast, the drawbacks of this method are that the tagging might disrupt the formation of protein complexes and a few of the proteins might not exist in the given situations and could be ignored [14]. Even though experimental approaches, for example, immune precipitation, generated great quality outcomes and these approaches have produced big volumes of interaction data, they were extremely time consuming and their outcomes of the high-throughput techniques contain a great number of false-negative and false-positive relationships [15]. In addition to experimental methods, computational methods can explain protein-protein interactions at various levels [16].

## Computational methods to detect PPI

Computational methods might emphasize thorough investigation or perform a wide scale examination across huge datasets. They might deduce whether proteins interrelate via protein sequence and genomic analysis. The approaches using protein sequence and genomic data contain a study of the absence or presence of genes in associated species, gene fusion events, preservation of gene neighborhood, interconnected mutations on surfaces of protein, the resemblance of phylogenetic trees, co-occurrence of sequence domains, functional and co-expression features [17]. Sometimes, integration of these features is used to predict new interactions or to approximate the validity of PPIs, which are evaluated experimentally [18]. Some features such as likeness in the Gene Ontology (GO) term annotation, co-expression, sequence and the existence of possibly interacting domains of the protein pair under many conditions or numerous tissues have been revealed to be applicable predictors of protein-protein interactions [19]. For the prediction of PPIs, physical docking methods recently were revealed to create good outcomes [20]. However, this technique is restricted by the computational complication and the tertiary configuration of the big number of proteins has not yet been identified [21].

## PPI datasets

Experimentally detected PPIs are collected in several publicly available databases that are curated by experts and make the PPI's supporting evidence easily available. Typically, these databases provide meta-data such as the study in which the interaction has been described and which techniques have been utilized to measure the interaction. These databases apply diverse mechanisms to display and query the data. These databases include HPRD [22], BioGRID [23], MINT [24] and IntAct [25]. It has been revealed that the human proteome takes account of about 300 000 PPIs out of a potential 4300 000 000 PPIs. This approximation does not represent the numerous variations in interacting pairs due to post-translational modifications and alternative splicing. A number of databases have been recognized and developed to fill this gap by predicting PPIs. Furthermore, human diseases and other traits are being probed by genome-wide screens. For example, several recent studies demonstrate genome-wide screening endeavors to recognize somatic mutations in several cancer types [26]. Location of genes or proteins into a pathway context can yield evidence about the links among these genes and has the potential to create hypotheses about the mechanism(s) of relating these genes to phenotypes [27]. Reliable pathway databases are essential for such an analysis. Overall, the prediction of PPIs by databases are based on various types of evidence including presence of fusion evidence, co-occurrence evidence, experiment evidence,text mining and coexpression evidence [28].Currently there are several existing protein-protein interaction databases that focus on experiment or predict evidence as exemplified in Table 1.

## PPI network

The network of interactions amid proteins is the skeleton that forms the properties of each living cell. Most processes rely on the ability of proteins to recognize and bind each other, whether it is enzymatic pathways or cascades of signal transduction. New experimental methods have enhanced attention on these networks, resulting in a fast growth in accessing data on protein interactions from numerous species. Some devices are needed to layout and display the network data, because of the great number of interactions present in PPI databases [45].

## Visualization Tools

Recently, in order to construction of protein-protein interaction network, different visualization tools have been developed. Table 2 shows the different tools and the access links.

These visualisation tools were compared based on essential features for protein-protein interactions analysis. Each visualisation tool has its strengths and limitations. Among these visualisation tools, Cytoscape is the most popular graph viewer for PPI network and is applied to analyze protein interaction information, expression and metabolic profiles. It contains several applications as plug-ins that make the software appreciable regarding various scientific purposes. Another superiority of Cytoscape among other visualization tools is the integration with several well-known databases such as IntAct, DIP, KEGG, etc. It allows one to represent even large PPI map of thousands of interactions. It performs several layout algorithms and demonstrates a wide range of interaction network analysis from basic to advanced options. In Cytoscape, a large number of plugins implement all types of functionality ranging from aforementioned PPI databases to high-level network algorithms. For instance, one of the important aspects of protein interactions analysis is the attributes and annotation of proteins that Cytoscape provides users to download annotations such as Gene Ontology (GO) [51].

NAViGaTOR is a simpler and more user-friendly tool that enables to visualize huge data groups as protein interaction network in 2D and 3D view. Its particular advantage is the ability to extract data directly from I2D [52] and cPATH [53].In addition, it allows data to be imported in BIOPAX, XML, GML, PSI-MI, and tab-delimited text format, which are the common formats to process protein interaction network. The common exported formats of the interaction network are SVG, PDF, JPEG, BMP, Pajek, and TIFF format. The protein-protein interactions that are represented in the network panel can be modified, for example it can be differentiated in shape or colour of the nodes. NAViGaTOR also enables to consider multiple network panels at the same time, therefore the multiple interaction networks can be compared. Furthermore, the protein nodes can be transferred from one interaction network to another by copy and paste. NAViGaTOR is also able to extract protein data from various databases such as GO directly and the retrieved data can be saved in the created protein interaction network. The network can be filtered and classified in different colours and node sizes automatically according to GO information after the GO info are inserted into the network. Proteins within a biological network can be subgrouped according to different functions or features.

Pajek is an older tool that is able to create 2D and pseudo 3D view for protein interaction network. Pajek is limited in integrating with any database and provides only flat file format that is not compatible with most of the XML formats. Therefore the achieved data from different databases should first be converted into Pajek file format and then imported to visualize. These limitations have restricted the utilization of Pajek by users.

Gephi is another visualization tool that is able to process huge data sets in 3D interaction network view. Similar to Cytoscape, Gephi also provides several applications, namely plug-ins to analyse the network toward different scientific purposes. However, as common PSI-MI files are not supported by Gephi, the imported file format should be converted to formats that are supported by Gephi. On the other hand, the outputs of various protein databases are not compatible with Gephi. These limitations make difficulties for users to apply Gephi. Biolayout Express 3D is a powerful network visualisation tool that enables users to map interaction network in 2D and 3D view. Although using of Biolayout is easy and useful in analysing large data sets, it does not integrate with protein databases and are not supported by plug-ins. Moreover, the customised modification of nodes is allowed but cannot be saved for future use.

Medusa is a simple, open source visualisation tool that is designed to construct protein-protein interaction networks from the STRING database [54].It provides 2D view for biological network and the advantage of this tool is its ability to change background images that can be inserted by users. It is a Java application and does not require installing onto an operating system. However, it is not able to analyse the huge data and is designed for analysing the small datasets.

Similar to Medusa, Arena3D is also a simple tool that does not require installation. The difference between these two tools is Arena3D projects network in multiple layers in a 3D space. This feature allows user to view biological networks in a less complex and more comprehensible way by classifying the proteins according to locations, diseases, structures and pathways in different layers. However, similar to Medusa, Arena3D possesses its own input file format, thus the saved data should be converted to the Arena3D supported file format. The comparison is summarised in Table 3. After taking all the strengths and limitations of each visualisation software into consideration, Cytoscape is judged to be the best as the main analysis tool throughout the study.

## Network topological analysis to discover essential proteins in PINs:

In a network, the interactions amongst proteins are exhibited in the formal context of graph theory. A network graph comprises a set of nodes and a set of edges that link the nodes. Several research questions associated with the function of single or groups of interacting proteins can be answered with the help of PPI networks [55]. Essential proteins for a biological event play a complex role in the development of process and discovery of their features is an interesting research subject in proteomics. A protein's essentiality has been used in numerous medical and biological researchers in recent years. Currently, essential proteins are recognized based on gene knockout experiments, which can be expensive and time consuming when the biological experiments are done on a largescale basis. Computational methods can supply the knowledge of social network analysis, graph mining and biological information. The absence or dysfunction of essential proteins would create an adverse disruption to the topological stability of the network as in the case of PIN biological lethality. This laid the foundation where computation methods based on topological features are developed to better detect essential proteins [56]. Protein-protein interaction networks of different species interestingly have many common topological features. PPI networks are also said to have a power-law degree distribution defined as: P(x) = Cx- α, where C = e and P(x) is a probability that a selected node has exactly x value (degree node). α is the degree exponent which defines some properties of the network. Degree exponent values for most of the known networks in nature are between two and three. It is recognized that networks with a degree exponent larger than three do not have features of scale-free networks [57].

## Four factors:

shortest paths, degree (connectivity), betweenness centrality (BC), and closeness centrality (CC), are established on the properties of each node in a PPI network and were adopted to analyze general mathematical properties of the PPI networks and to search topologically important and essential proteins [58]. Degree (or connectivity) informs how many links a node has to other nodes and the degree dissemination is acquired via counting the number of nodes with a specified degree and dividing by the total number of nodes. The degree distribution discloses comparatively fewerstrongly associated nodes, which are branded as hubs, and they play a key role as a local property in the network [59].

Betweenness centrality (BC) was computed to get non-hub proteins which still play significant parts as a global property, as the BC is a valuable tool for identifying bottlenecks in a network. For node k, BC is described as:

b(k) = Σ_i_,_j_bi→(k) = Σ_i_,_j_(g^k^_i_→_j_ / g_i_→_j_),

where gi→j is the number of shortest geodesic paths from node i to node j, and g^k^_i_→_j_ is the number of geodesic paths among g_i_→_j_from node i to node j that cross node k [60]. Another significant aspect of bottleneck protein nodes and hub is that they are prospective drug targets.

Closeness centrality (CC) is the opposite of the network diameter, described as the medium number of hops (jumps) via the shortest geodesic paths from node k to all other nodes. The diameter symbolizes the capability of two nodes to interconnect with each other: if the diameter (the larger CC) is smaller, the predictable path between them will be shorter. Thus, a big CC shows that the node is near to the topological center of the network [61]. By computing the length of all the geodesics from or to the vertices in the network, the shortest path (geodesics) is calculated. In order to see how many average steps were needed, the average shortest path was computed, to connect two randomly chosen nodes in the network[62].

## Functional analysis, clustering and drug discovery:

Proteins usually do not function alone but carry out their task with assistance from other proteins. Functional analysis represents functional groups of the protein that are involved in a protein interaction network. A common analysis of PPI networks is to identify the unknown function of a protein according to the known functions of its interaction partners. The underlying presumption is based on the states that two proteins that interact likely share a common function (9). This principle underlies many protein annotation tools. For example, the popular gene function prediction tool GeneMANIA is implemented as a web server and a Cytoscape plugin [63]. ClueGo program allows us to integrate several ontology sources because in each source, for each gene, there is a large amount of information. ClueGo can extract the nonredundant biological information for a large cluster of genes using GO, KEGG, BioCarta, REACTOME and Wiki Pathways. Functional network is an interaction network that represents functional relationship between the nodes of the network. Network modules recommend that the contributing proteins perform together closely, for instance in cellular pathways or protein complexes.Therefore, the modular organization of large PPI networksis exploited by numerous methods to envisage proteins that act together in functional sub networks. The identification of groups of proteins that closely interact has been made possible by many network clustering tools. With high clustering coefficients, generally a big network is looked over for modules and more interactions are molded inside the module than to proteins outside the module. Within a clique, a maximum coefficient is attained that is an entirely linked graph neighborhood and Cytoscape plugin. For example, Allegro MCDE is a graph clustering algorithm which is capable of efficiently identifying these structures [64].

Functional protein interaction networks of several diseases, namely "Network Pharmacology" as a novel approach is applied to study disease network such as Alzheimer's disease [65], cancer [66], and metastasis [67] by constructing and analyzing the protein interaction network using wet-lab data derived from the protein interaction databases [68]. The analysis of the network pharmacology can help in the study of drug discovery and to better understand their possible side effects and toxicity, because the protein-targets do not function alone and carry out their task in connectivity with other proteins [69,70]. Protein interaction approach and topological analysis of the network has been applied to discover drug targets for treatment leishmania infection [71].

Protein networks have been applied in order to compare "disease" versus "normal" states and also to determine general characteristics of the proteins involved in disease [72]. Constructing and analyzing the protein network associated with several diseases can help to find new proteins involved in disease progress. Some studies have revealed that disease proteins are more interconnected than nonessential proteins in protein networks [73]. Other studies have shown that the proteins neighbor to disease proteins tend to interact with other proteins associated with that disease [74]. Further attempts have also shown that connectivity changes in the protein interaction network from healthy to diseased states can be valuable for predicting novel appropriate drug targets [75,76].

Disease network could explain the important molecular function of the disease in order to find the potential drug targets. In a disease network, the ideal drug target must be essential in diseased cell and inhibiting their function should be less knotty in the whole functional system. Accordingly the potential targets are placed in a strategic point in the disease [77]. Using biological network, different algorithms and methods are being developed in order to identify potential drug targets. Some of these approaches provide quantitative analysis to recognize essential proteins for the "information flow" within a disease network [78]. Other strategies identify nodes as potential drug targets that block a specific pathway, but do not affect other processes; these targets rewire their signaling network using modular protein switches. Some methods try to identify the ideal drug targets from the standpoint of efficacy and side effects. In this way, the nodes, namely "bridging nodes", are those nodes in the network which are less essentially involved in connecting or bridging modular sub regions of a network and may be potential targets [79]. Other approaches are also investigated for pathogen cases in order to remove a pathogen. The targets for these diseases are hub proteins of the pathogen interaction, which are lost in the host organism [80]. Some disease mechanisms can affect multiple genes or can bypass the block of a single target. Therefore, the identification of multiple targets for these diseases network is necessary. The most common carcinomas will not be treated by simple targets and the understanding of involute mechanisms is highly required to study their inhibition through a combination of drugs. In these cases, it has been reported that the partial interruption of an interesting small number of targets can be more affected than the full inhibition of a single target [81].

## Assess the quality of molecular interaction:

As described above, the PPIs are detected by a high- throughput technology like Y2H or TAP/MS, Small- Scale single protein studies, or computational predictions. Their outcomes contain a great number of false-negative and false-positive relationships. Therefore, assigning confidence score to individual interaction is a requirement for quality assessment of the interactions.

## Confident experimental and physical interactions:

MINT is a PPI database that provides a score that represents the reliability of each interaction based on a heuristic integration of the available evidence into combined experimental evidence [24]. To derive a high-confidence network of literature-curated interactions, protein complexes from iRefWeb were converted into pairwise interactions using matrix expansion and MINT-inspired score was used to determine high-confidence pairs. The represented MINTinspired score was assigned based on MINT (MI) score, and for detecting the high confidence of PPIs, the following procedure has been applied; 1) Take all relevant protein-interaction pairs from iRefWeb, whether from binary interactions or from the matrixexpansion of complexes; 2) Exclude interactions that are supported by less than 3 publications or are not conserved in any species; 3) Retain pairs with an MI-score of at least 0.431 [82].

## Confident predicted and functional protein interactions:

PPIs were built using six separate prediction parameters: Neighborhoods, Co-occurrence (phylogenetic profiles), Fusion, Coexpression, Experimental Interactions, and Text-mining. Each of these parameters has its own score (raw) of measurements such as intergenic distances, Euclidean distances, fusion z-score, Pearson correlation coefficient, various experimental score (e.g. qualitative binary score), and log-odds score. Each raw score was benchmarked using the KEGG database. PPIs that occurred on the same metabolic KEGG map were considered to be true positive and those that occurred on a different map were not. Due to the sigmoidal correlation between raw score and fraction of PPIs on the same KEGG map, STRING fits those correlations to the hillequation to derive the confidence score. STRING derived scores correspond to the probability of finding the PPI within the same KEGG pathway or map [54]. Different scores on the same bench mark provide a platform of comparisons among the scores and equivalent scores can be calculated. This equivalency mapping helps to combine the scores into a single score, which express higher confidence and gives higher coverage (number of predicted PPI) at a specific accuracy. STRING uses a score combiner based on the product of probabilities using the following formula:

 S = 1-∏^N^_i_(1 - S_i_)

with Si the probability score for database i, S the combined score and N the total number of databases to be combined. The combined scores were further rescaled into the confidence range from 0.0 to 0.1 combining all the scores. Those indicate: <0.400 (low confidence), 0.400-0.700 (medium confidence) and >0.700 (high confidence) [83].

## Conclusion:

Discovery of the protein connections is critical to understand the cellular pathway. Due to the difficulties in analyzing vivo PPI's, the protein interaction databases and computational tools are being developed to construct and analyze protein interaction networks. New protein detection and drug target discovery regarding therapeutic strategies are conceivable, surprisingly, through indepth analysis of the network pharmacology and disease networks.

## Figures and Tables

**Table 1:** Protein interaction databases

Databases	Descriptions	References and URL
The Database of Interacting Proteins (DIP)	DIP is a database that records experimentally determined protein-protein interactions. It provides the scientific community with an integrated set of tools for browsing and extracting information about protein interaction networks. Tools have been developed that allow users to analyse, visualize and integrate their own experimental data with the information about protein-protein interactions available in the DIP database.	[29] http://dip.doe-mbi.ucla.edu/dip/Main.cgi
IntAct	IntAct is an open source database suite for storing and analysing protein-protein interaction data. The available data emanates from published literature and is manually interpreted by expert biologists to a high confidence of detail, comprising experimental methods, conditions and interacting domains. The experimental methods include yeast-2-hybrid, mass spectrometry, fluorescence microscopy, co immune precipitation, pull down and others. PPI network data can be import from IntAct directly using IntAct Web Service Client, a plugin of Cytoscape.	[30] http://www.ebi.ac.uk/intact/
The Biomolecular Interaction Network Database (BIND)/(BOND)	BIND (/BOND) is a database designed to store full descriptions of interactions, molecular complexes and pathways. It can be used to study networks of interactions, to map pathways across taxonomic branches and to generate information for kinetic simulations.	[31] http://bond.unleashedinformat ics.com/Action?
A Molecular Interaction database (MINT)	MINT is a database designed to store functional interactions data such as enzymatic modifications of one of the partners. MINT includes of extracted data from the published literature by expert curators and software that assemble abstracts comprising information from interaction and demonstrated them in a user-friendly format. The interaction data can be easily mined and observed graphically through 'MINT Viewer'.	[32] http://mint.bio.uniroma2.it/mint/Welcome.do
Reactome	Reactome is a peer-reviewed resource of human biological pathways. The complete set of possible reactions organizes its reactome by enter the genetic profile of an organism. Reaction is the basic module of the Reactome database then the reactions are grouped into causal strings to procedure pathways. The involving applications have been developed to enter custom data and interpretation by expert biologists, and to allow visualisation to construct an interactive pathway network.	[33] http://www.reactome.org/
Human Protein Reference Database (HPRD)	HPRD is an open source based on technologies for protein features in various aspects of human proteins comprising post-translational modifications enzyme-substrate links, disease associations and PPI. The details were derived manual accurate reading of the scientific literature by expert biologists and also protein sequence analyses by bioinformatics approaches.	[34] http://www.hprd.org/
MIPS	The MIPS or mammalian protein-protein interaction database (MPPI) is a new high-quality resource which stores experimental protein interaction data in mammals. The data is based on published experimental studies that has been analysed by human expert curators. It provides a flexible and powerful web interface with full dataset for download toward various scientific targets.	[35] http://mips.helmholtz-muenchen.de/proj/ppi/
SMART (a Simple Modular Architecture Research Tool)	SMARTanalysis the domain structures of proteins and provides the documentation and annotation of genetically the domains. The domains which are grouped in more than 500 domain families are widely annotated in order to phyletic distributions, tertiary structures, functional class and functionally important residues. Each domain found in a non-redundant protein database as well as search features are collected in an associated database system.	[36] http://smart.embl-heidelberg.de/
Kyoto Encyclopaedia of Genes and Genomes (KEGG)	KEGG is the reference knowledge base that integrates current knowledge on molecular interaction networks such as pathways and complexes, information about genes and proteins generated by genome projects and information about biochemical compounds and reactions.	[37] http://www.genome.jp/kegg/
InterPro	InterPro is a resource that provides functional analysis of protein sequences by classifying them into families and predicting the existence of domains and main sites. To organize proteins in this way, InterPro procedures predictive models, represented by several different databases. The databases that make up the InterPro Consortium contain PROSITE, HAMAP, Pfam, PRINTS, ProDom, SMART, TIGRAFAMs, PRISF, SUPERFAMILY, CATH, Gene30 and PANTHER.	
Gene Ontology (GO)	The GO Consortium is the set of model organism and protein databases and biological research communities actively involved in the development and application of the Gene Ontology.	[39] http://geneontology.org/page/go-database
BioGRID	BioGRID is an online interaction repository with data compiled through comprehensive curation efforts. This database contains protein and genetic interaction from major model organism species. All interaction data are freely provided via search index and available by downloading in a wide variety of standardized format.	[40] http://thebiogrid.org/
Pathway Commons	Pathway Commons collects publicly available pathway information from various organisms. It allows convenient access to a comprehensive store of biological pathways from multiple resources presented in a common language for gene and metabolic pathway analysis.	[41] http://www.pathwaycommons.org/about/
BioCyc	The BioCyc is a collection of genomes and metabolic pathways which are represented by multiple pathways. The included data is generated by software that predict the metabolic pathways of completely sequenced organisms. BioCyc also integrates protein feature and Gene Ontology information from other bioinformatics databases, such as from UniProt.	[42] http://biocyc.org/
Pfam	The Pfam database provides a large collection of protein families, each output results by multiple sequence alignments and hidden Markov models (HMMs).	[43] http://pfam.xfam.org/
GEO (Gene Expression Omnibus)	GEO is an international public source that stores freely microarray, next-generation sequencing, and other forms of high-throughput functional genomics data submitted by the research community. GEO fallows three main aims: archive high-throughput functional genomic data; collect and well-annotated data from the research community; provide to researchers to query, review and download gene expression profile of interest.	[44] http://www.ncbi.nlm.nih.gov/geo/

**Table 2:** Common tools for visualization protein interaction network

Visualization tools	URL
Cytoscape [46]	http://www.cytoscape.org/
NAViGaTOR [47]	http://ophid.utoronto.ca/navigator/
Biolayout [48]	http://www.biolayout.org/
Medusa [49]	https://sites.google.com/site/medusa3visualization/
Arena3D [50]	http://www.arena3d.org/

**Table 3:** Comparisons of Visualization Tools in Several Features Including Database Integration, Data Input Format, Export File Format, and Layout Algorithm

Features	Arena3D	Biolayout	Cytoscape	NAViGaTOR	Pajek	Gephi	Medusa
Plug-ins	x	x	√	x	x	√	x
Auto merge numbers of networks (no modification on original input file)	x	x	√	√	x	x	x
Mark nodes according to GO annotations	x	x	√ (plug-ins)	x	x	x	x
GO annotations	x	x	√ (plug-ins)	√	x	x	x
							
Database Integration							
IntAct	x	x	√ (plug-ins)	x	x	x	x
NCBI	x	x	√ (plug-ins)	x	x	x	x
APID2NET	x	x	√ (plug-ins)	x	x	x	x
cPath	x	x	√ (plug-ins)	√	x	x	x
GO	x	x	√ (plug-ins)	√	x	x	x
							
Data Input Format							
Text delimited data	x	√	√	√	x	x	√
GML	x	x	√	√	x	√	x
PSI-MI XML	x	x	√	√	x	x	x
SIF	x	√	√	x	x	x	x
BIOPAX	x	x	√	√	x	x	x
XGMML	x	x	√	x	x	x	x
Text	√	√	√	√	x	x	√
							
Export File Format							
Image file	√	√	√	√	√	√	√
xml		x	x	√	x	x	x
Pajek	√	x	x	x	√	x	√
gml	x	x	√	√	x	x	x
PSI-MI	x	x	√	√	x	x	x
Arena3D	√	x	x	x	x	x	√
text	√	x	x	√	x	x	x
sif	x	√	√	x	x	x	√
svg	x	x	√	√	√	√	x
tiff	x	x	x	√	x	x	x
pdf	x	x	√	√	x	√	x
GraphViz	x	x	x	x	x	x	√
Medusa format	√	x	x	x	x	x	√
VRML	√	x	x	x	√	x	x
							
Layout Algorithm							
Multi-threaded	x	x	x	√	x	x	x
grid-variant layout algorithm							
Spring Embedded algorithms	√	√	√	x	√	x	√
Fruchterman-Rheingold layout algorithm	√	√	x	x	√	x	√
Lanczos algorithm	x	x	x	x	√	x	x
Force Atlas algorithm	x	x	x	x	x	√	x
Distance Geometry layout	√	x	x	x	x	x	√
Simulated Annealing Algorithm	√	√	√	x	x	x	x
Circular layout	√	x	√	√	√	√	√
Hierarchiral layout	√	x	√	x	x	x	√
Yifan's Hu Multilevel layout	x	x	x	x	x	√	x
